# Distribution and morphology of nitrergic neurons across functional domains of the rat primary somatosensory cortex

**DOI:** 10.3389/fncir.2012.00057

**Published:** 2012-11-06

**Authors:** Anaelli A. Nogueira-Campos, Deborah M. Finamore, Luis A. Imbiriba, Jean C. Houzel, João G. Franca

**Affiliations:** ^1^Laboratório de Neurobiologia II, Programa de Neurobiologia, Instituto de Biofísica Carlos Chagas Filho, Universidade Federal do Rio de JaneiroRio de Janeiro, Brazil; ^2^Departamento de Fisiologia, Instituto de Ciências Biológicas, Universidade Federal de Juiz de ForaJuiz de Fora, Brazil; ^3^Escola de Educação Física e Desportos, Universidade Federal do Rio de JaneiroRio de Janeiro, Brazil; ^4^Laboratório de Fronteiras em Neurociências, Programa de Ciências Morfológicas, Instituto de Ciências Biomédicas, Universidade Federal do Rio de JaneiroRio de Janeiro, Brazil

**Keywords:** NADPH-diaphorase, dendritic morphometry, nitric oxide, inhibitory neurons, rat cortical area S1, barrel cortex

## Abstract

The rat primary somatosensory cortex (S1) is remarkable for its conspicuous vertical compartmentalization in barrels and septal columns, which are additionally stratified in horizontal layers. Whereas excitatory neurons from each of these compartments perform different types of processing, the role of interneurons is much less clear. Among the numerous types of GABAergic interneurons, those producing nitric oxide (NO) are especially puzzling, since this gaseous messenger can modulate neural activity, synaptic plasticity, and neurovascular coupling. We used a quantitative morphological approach to investigate whether nitrergic interneurons, which might therefore be considered both as NO volume diffusers and as elements of local circuitry, display features that could relate to barrel cortex architecture. In fixed brain sections, nitrergic interneurons can be revealed by histochemical processing for NADPH-diaphorase (NADPHd). Here, the dendritic arbors of nitrergic neurons from different compartments of area S1 were 3D reconstructed from serial 200 μm thick sections, using 100x objective and the Neurolucida system. Standard morphological parameters were extracted for all individual arbors and compared across columns and layers. Wedge analysis was used to compute dendritic orientation indices. Supragranular (SG) layers displayed the highest density of nitrergic neurons, whereas layer IV contained nitrergic neurons with largest soma area. The highest nitrergic neuronal density was found in septa, where dendrites were previously characterized as more extense and ramified than in barrels. Dendritic arbors were not confined to the boundaries of the column nor layer of their respective soma, being mostly double-tufted and vertically oriented, except in SG layers. These data strongly suggest that nitrergic interneurons adapt their morphology to the dynamics of processing performed by cortical compartments.

## Introduction

The cortical nitrergic system is composed of inhibitory GABAergic neurons that express nitric oxide synthase (NOS) (Bredt et al., 1991; Dawson et al., 1991; Hope et al., 1991; Yan and Garey, 1997; Kubota et al., 2011), the enzyme responsible for synthesis of nitric oxide (NO). NO is a highly diffusible gas with a short half-life (0.5–5 s), whose range of action covers about 100–200 μm, rising within 10–15 s to a steady-state concentration (Malinski et al., 1993; Meulemans, 1994; Wood and Garthwaite, 1994). Thus, NO could spread out from its site of production to influence many different tissue elements (neuronal, glial, and vascular components) that are not necessarily in close anatomical juxtaposition (review in Garthwaite and Boulton, 1995). NO is an unusual neurotransmitter (Bredt et al., 1991; Dawson and Snyder, 1994), involved in a wide range of physiological and pathological events in the central nervous system, such as modulation of synaptic transmission and neurotoxicity (Wallace et al., 1996; Estevez et al., 1998; see Calabrese et al., 2007; Garthwaite, 2008 for review). NO has been associated with learning and memory in addition to neurovascular coupling (Bohme et al., 1991; Izumi et al., 1992; Iadecola, 1993). Activation of NOS is one of the factors that leads to vasodilation following neuronal activation, leading to an increase in local cerebral blood flow (Drake and Iadecola, 2007; Cauli and Hamel, 2010). Nitrergic neurons are part of the intrinsic neuronal circuitry. They release neuropeptide Y (NPY)—a potent vasoconstrictor—on blood vessels located distal to the cell body. Distal release of NPY may restrict the NO-induced vasodilation to the micro region around the nitrergic neuronal cell body and dendrites (Estrada and DeFelipe, 1998; Cauli et al., 2004).

NADPH-diaphorase (NADPHd) is a generic term attributed to any enzyme that chemically reduces the co-factor NADP. Action of fixatives, especially paraformaldehyde, deactivates most endogenous diaphorases, except for NOS, rendering this enzyme detectable by histochemistry in fixed tissue (Matsumoto et al., 1993). Thus, histochemistry for NADPHd became a reliable tool for the study of the distribution of NOS in the nervous system. In the mammalian cerebral cortex, the population of NADPHd-stained neurons has been subdivided into two broad categories according to the intensity of histochemical staining and soma size. Type 1 neurons present intense Golgi-like staining of the cell body and dendrites, and large somas. Compared to type 1, type 2 neurons are smaller with lighter labeling of the cell body and, occasionally, some labeling in primary neurites (Sandell, 1986; Bredt et al., 1990; Dawson et al., 1991; Vincent and Kimura, 1992; Lüth et al., 1994; Yan and Garey, 1997; Iwase et al., 1998; Franca et al., 2000; Freire et al., 2004). Type 1 neurons comprise about 0.5–2% of all cortical neurons (Gabbott and Bacon, 1995) and are much less frequent in neocortex than type 2, which display a different laminar distribution (Cruz-Rizzolo et al., 2006). Finally, in addition to individual neurons, histochemistry for NADPHd also labels very thin neuronal processes that can be detected as a diffuse neuropil stain, especially along the thalamo-recipient layer IV. NADPHd-reactive neuropil depicts boundaries of cortical primary sensory areas in different species (Franca et al., 2000; Freire et al., 2012); and can additionally allow delineation of cortical modules in somatosensory cortex of rodents (Franca et al., 2000; Pereira et al., 2000; Freire et al., 2005, 2012) and in visual cortex of primates (Sandell, 1986; Franca et al., 1997).

Primary somatosensory area (S1) in the rat provides a useful model system for exploring details of cortical organization in mammalian brains (Petersen, 2007) because it presents generalized features, such as (1) a distinct laminar arrangement in six different parallel layers from the pia mater to the white matter; (2) a representation of sensory surfaces that form a topologically organized map (Santiago et al., 2007); and (3) a vertical organization with sharply defined anatomical units, the so-called “barrels” (Woolsey and Van der Loos, 1970, reviewed in Catania, 2002) that can be depicted by NAPDHd histochemistry (Franca and Volchan, 1995).

In rodent S1, recent studies on the distribution and morphology of nitrergic neurons (type 1 NAPDHd neurons) were mainly performed on tangential sections along layer IV of the posterior medial barrel subfield (PMBSF)—the region where mystacial vibrissae are represented and the barrels are large and very sharply-defined. Such studies revealed that nitrergic neurons concentrate in septal domains of the PMBSF (Valtschanoff et al., 1993; Franca and Volchan, 1995; Pereira et al., 2000; Freire et al., 2004, 2005), where they display larger and more complex dendritic trees than neurons located inside barrels (Freire et al., 2005). Nonetheless, the PMBSF does not represent the entire rodent S1 since additional barrel fields, representing the anterior snout, the forepaw, the hindpaw, and the trunk, plus an intervening “dysgranular” cortex in-between the barrel fields, are also part of this cortical area (Wallace, 1987). Additionally, S1 is composed of different cortical compartments representing different cortical layers in different processing columns (*i.e.*, barrel and septal columns) comprising different components of S1 cortical circuit (Lubke and Feldmeyer, 2007; Wester and Contreras, 2012). So far there is no description of the distribution and fine morphological arrangement displayed by the dendritic arbors of these cells along the entire S1, nor about how these neurons might relate to the barrel cortex architecture. While being both volume diffusers for NO and inhibitory (GABAergic) elements of cortical circuits, type 1 NAPDHd neurons might display different dendritic arrangements according to their position in the cortical circuitry.

Thus, after subdividing rat S1 into six basic compartments—supragranular (SG), granular (GR), and infragranular (IG) layers located in barrel or septal columns—we first quantified the distribution of nitrergic neuronal cell bodies in these compartments, and then studied the morphology and spatial arrangements of their dendritic trees. We observed that dendrites from single nitrergic neurons can cross cortical compartments, not being restricted by compartment borders. Septal columns tended to present higher concentration of nitrergic neurons than barrel columns. Additionally, in SG layers nitrergic neuronal density is the highest among laminar compartments. Dendritic fields in SG assumed various spatial organizations, being multipolar, horizontal or vertical. In contrast, most nitrergic neurons displayed vertically-oriented dendritic arbors in GR and IG layers. We propose that these subtle differences in the morphological organization of local nitrergic neurons reflect different spatial characteristics of the neurovascular coupling between local hyperemia and neuronal excitability that allow distinct processing dynamics in each cortical compartment.

## Materials and methods

### Animals

The left hemispheres of three adult male Wistar rats were used in the present study. All efforts were made to minimize animal suffering and to reduce the number of animals used. Protocols for these experiments were approved by the Ethic Committee for Animal Use in Scientific Research of the Centro de Ciências da Saúde (CCS, Universidade Federal do Rio de Janeiro) and were in accordance with the NIH guidelines (assurance number “IBCCF 029”).

### Perfusion and histological procedures

Animals were deeply anesthetized by inhalation of ether vapor and perfused through the left ventricle, using a peristaltic pump, with 300 ml of 0.9% sodium chloride (NaCl), followed by 300–400 ml of 4% paraformaldehyde in pH 7.4, 0.1 M sodium phosphate buffer (PB). After removing the brain from the skull, a block containing the entire parietal lobe was prepared and cut coronally with a vibratome (Pelco International, Series 1000) into serial, 200 μm-thick sections. The sections were collected in PB and subsequently washed three times in this same solution. Next, the sections were incubated free-floating at 37°C in a solution for NADPHd histochemistry, consisting of 0.6% malic acid, 0.03% nitroblue tetrazolium, 1% dimethylsulfoxide, 0.03% manganese chloride, and 0.5% β-NADP in 0.1 M Tris buffer, pH 8.0 (modified from Scherer-Singler et al., 1983). To increase penetration of reagents into the section thickness, the detergent Triton X-100 was added to the histochemical solution in a concentration of 1.5–3%. This concentration has been used for adequate penetration of NADPHd reagents through thick histological sections (Franca et al., 1997).

The reaction was monitored under light microscope, and usually interrupted after 2–4 h by rinsing the sections in PB. All procedures, from perfusion to NADPHd histochemistry were performed at the same day. Sections were then mounted onto gelatin-coated glass slides and left to air-dry overnight. Slides were dehydrated in alcohol, washed twice in xylene for 5 min and coverslipped with Entellan (Merck).

### Definition of S1 compartments and tridimensional reconstructions

In the rat, neuropil staining by NADPHd histochemistry allows the definition of boundaries of barrels and cortical layers in sections through S1 (Franca and Volchan, 1995). This cortical area can thus be subdivided in six cortical compartments corresponding to two vertical domains (barrel or septal column) and three horizontal compartments corresponding to SG layers, including layers I, II, and III; GR, corresponding to layer IV, where barrels and septa were most evident; and IG layers, including layers V and VI (for an example, see Figure 4).

All coronal sections through S1 were analyzed and reconstructed using a Zeiss Axioplan-2 microscope equipped with a color digital camera (1600 × 1200, 3/4” chip, 36bit, MBF), a motorized stage (Mac5000 LUDL), and an extra *z* encoder controlled by Neurolucida software (MBFBiosciences, Inc) running on a Dell workstation. Cortical boundaries and barrel contours defined by neuropil reactivity, and the relative position of S1 strongly reactive (type 1) NADPHd cell bodies (Yan and Garey, 1997) were digitized using 5x and 10x objectives, respectively. Individual neuronal profiles were three-dimensionally (3D) reconstructed using a 100x oil objective, and included both the contours of the cell body and the entire dendritic tree as apparent in the cortical section in which the cell body was contained.

Type 1 neurons in area S1 were selected for morphometric analysis based on apparent completeness of their dendritic trees. Only neurons displaying dendritic trees characterized by a tapering profile of all dendrites, with none of them ending abruptly in a stump, were selected for reconstruction. In each of the three hemispheres, 180 neurons were reconstructed, corresponding to 30 neurons for each of the six cortical compartments defined above. In hemisphere R06-10, all S1 type 1 neurons were reconstructed in all sections for qualitative appraisal of dendritic tree distribution, but only 180 of these neurons were chosen for morphometric analysis, based on the criteria described above.

### Data extraction and statistical analysis

#### Definition and measurement of cortical compartment areas

Each barrel column was defined by a radial projection from the lateral borders of each layer IV barrel to the pial surface and the layerVI/white matter border (Figure 4). The area of the barrel column was then measured using ImaqVision 6.0 image processing software (ACD Systems). The total area of septal columns was obtained by subtracting the area occupied by barrel columns from the total area of S1. Similarly, the area occupied by SG, GR, and IG layers were also measured.

#### Stereological evaluation of cell density

In order to estimate the density of type 1 NAPDHd neurons in each compartment volume (*N*_v_), we initially counted the number of cell body profiles (*N*) and divided it by the flat area (*A*) of the cortical compartment. Density per volume was then estimated for each cortical compartment adopting Abercrombie's stereological correction formula (Abercrombie, 1946):

$$N_{\text{v}}=\left(\text{N}/\text{A}\right)/\left(\text{t}+\text{d}\right)$$

where *t* was the section thickness, and *d* was the average cell body diameter in each compartment estimated by the procedures described by Schuz and Palm (1989). We assumed that *t* was constant, corresponding to the thickness set at the vibratome (200 μm). To calculate *d*, we first estimated *d* from the cell body area (*a*), using the formula of the circle:

$$d=\;2{\left(\text{a}/\pi \right)}^{\text{0},\text{5}}$$

The average cell body diameter (*d*) was calculated for each cortical compartment using all reconstructed neurons located in that given compartment. Then, the value of *d* was corrected using the stereological estimations defined by formulae (2) and (3) of Schuz and Palm (1989); yielding to values *d*_2_ and *d*_3_, respectively. Since *d*_2_ and *d*_3_ respectively overestimates and underestimates the actual value of *d* (Schuz and Palm, 1989), the final value of *d* applied in Abercrombie's formula was the mean obtained from *d*_2_ and *d*_3_. Values obtained for each compartment in each hemisphere are listed in Table 1.

**Table 1:** **Distribution of nitrergic neuronal cell bodies in the different cortical compartments**.

Cortical compartments^*^	**No. of cells (%)**	**Compartment area in mm**^**2**^ **(%)**	**Cell body diameter (variance) in μm**	**Neuronal density in cells/mm**^3^^**^
**R06-10 (*n* = 27 SECTIONS)**				
SG	559 (26.3)	26.31 (19.6)	12.35 (3.43)	100
GR	268 (12.6)	26.67 (19.9)	13.07 (2.97)	47
IG	1298 (61.1)	81.00 (60.5)	12.32 (3.03)	75
BARRELS	1390 (65.4)	93.28 (69.6)	12.42 (3.05)	70
SEPTA	735 (34.6)	40.71 (30.4)	12.43 (3.43)	85
**Total**	**2125 (100)**	**133.98 (100)**	**12.42 (3.18)**	**75**
SG - barrels	357 (16.8)	19.23 (14.3)	12.30 (3.16)	86
SG - septa	202 (9.5)	7.09 (5.3)	12.44 (3.91)	128
GR - barrels	184 (8.6)	16.54 (12.4)	13.15 (3.07)	50
SR - septa	84 (3.9)	10.13 (7.6)	12.88 (2.75)	38
IG - barrels	849 (39.9)	57.51 (42.9)	12.31 (2.88)	66
IG - septa	449 (21.1)	23.49 (17.5)	12.33 (3.31)	86
**R07-03 (*n* = 22 SECTIONS)**				
SG	421 (27.0)	20.65 (19.3)	14.74 (4.40)	95
GR	284 (18.2)	23.02 (21.5)	16.0 (3.71)	57
IG	853 (54.8)	63.28 (59.2)	14.31 (2.59)	63
BARRELS	951 (61.0)	70.98 (66.4)	15.08 (4.28)	62
SEPTA	607 (39.0)	35.97 (33.6)	15.0 (3.83)	78
**Total**	**1558 (100)**	**106.95 (100)**	**15.0 (4.04)**	**68**
SG - barrels	258 (16.5)	14.14 (13.2)	15.0 (5.69)	86
SG - septa	163 (10.5)	6.51 (6.1)	14.57 (3.21)	128
GR - barrels	154 (9.9)	11.49 (10.7)	15.72 (3.69)	50
SR - septa	130 (8.3)	11.52 (10.8)	16.28 (3.68)	38
IG - barrels	539 (34.6)	45.34 (42.4)	14.64 (3.12)	66
IG - septa	314 (20.2)	17.95 (16.8)	14.0 (1.93)	86
**R07-04 (*n* = 25 SECTIONS)**				
SG	498 (32.6)	24.23 (20.5)	14.04 (2.23)	96
GR	305 (19.9)	23.73 (20.1)	14.90 (3.01)	60
IG	726 (47.5)	70.06 (59.4)	14.26 (2.16)	48
BARRELS	1018 (66.6)	70.52 (59.7)	14.41 (2.62)	67
SEPTA	511 (33.4)	47.50 (40.3)	14.39 (2.57)	50
**Total**	**1529 (100)**	**118.01 (100)**	**14.40 (2.58)**	**60**
SG - barrels	316 (20.7)	14.28 (12.1)	14.29 (1.92)	103
SG - septa	182 (11.9)	9.95 (8.4)	13.78 (2.47)	85
GR - barrels	206 (13.5)	12.38 (10.5)	14.72 (3.17)	77
SR - septa	99 (6.5)	11.35 (9.6)	15.09 (2.89)	41
IG - barrels	496 (32.4)	43.86 (37.2)	14.21 (2.78)	53
IG - septa	230 (15.0)	26.19 (22.2)	14.30 (1.62)	41

* Cortical compartments: SG, supragranular layers; GR, granular layer (layer IV); IG, infragranular layers.

** Corresponding values were calculated after stereological corrections described in section “Stereological Evaluation of Cell Density”.

#### Morphological parameters analyzed

A number of quantitative morphological parameters were extracted by NeuroExplorer software (MBFBiosciences, Inc) from the reconstructed type 1 NADPHd neurons. These parameters could be either related to the cell body or to the dendritic tree.

Cell body parameters included the cell body area (corresponding to the flat 2D surface occupied by the neuronal soma) and form factor. Form factor describes how spherical the cell body is. As the contour shape of cell body approaches that of a perfect circle it tends to a maximum value of 1. In contrast, as the contour shape flattens out, this value approaches a minimum of 0.

Morphological parameters related to the dendritic tree included: (1) number of first order dendrites; (2) dendritic length (DL), corresponding to the sum of the length of all segments of the dendritic tree; (3) number of nodes (each node corresponding to a point of dendritic branching originating two or more dendritic segments); (4) number of dendritic segments (each segment corresponding to a dendritic branch between two nodes, or between a node and the cell body, or to a terminal branch); (5) fractal dimension; and (6) area of the dendritic field (Wässle and Boycott, 1991). The fractal dimension gives a quantitative estimation of the complexity of the dendritic tree, describing the way the dendritic tree fills the area that comprised the dendritic field. The area of the dendritic field was accessed by convex hull analysis, which measures the size of the dendritic field by interpreting a branched structure as a solid object in a given volume or area.

Additionally, dendritic field orientation of each neuron was defined using wedge analysis of DL. A Cartesian coordinate reference frame was centered at the cell body, with its principal axis oriented toward the pia mater, and dividing eight equiangular wedges. The summed length of dendritic segments contained in each wedge (DL_*n*_) was measured to calculate a verticality index (*vi*) in which the sum of DLs in the four wedges close to the vertical axis was divided by the sum of DL measured in all eight wedges (total DL). The *vi* allowed the classification of type 1 neurons as horizontal- (*vi* = 0 − 0.32), multipolar- (*vi* = 0.33 − 0.65) or vertical-oriented (*vi* = 0.66 − 1). For vertically-oriented neurons, a dendritic directionality index (*di*) was calculated by subtracting DLs from the two wedges close to the white matter from DLs obtained in the opposite two wedges opening toward the pia, and dividing the result by the sum of DLs for those four wedges. This *di* further characterized whether vertically-oriented neurons were double-tufted (*di* = −0.50 *to* +0.50), or if their dendritic trees were oriented toward the pia (*di* = +0.51 *to* +1.0) or the white matter (*di* = −1.0 *to* −0.49).

#### Statistical analysis

Descriptive statistical analysis was used to characterize the cases, based on the mean value, standard deviation (SD) and coefficient of variation (CV) [(SD/mean)] for each parameter. Moreover, reconstructed neurons were grouped according to the compartment occupied by their respective cell body. All morphological parameters were compared employing Two-way analysis of variance (ANOVA) for repeated measures to access differences among each laminar (SG, GR, and IG) and columnar (septal *versus* barrels) compartments. Fisher *post-hoc* analysis was employed when significance was attained. Tests of normality were performed to determine the probability that the sample came from a normally distributed population (Shapiro–Wilk's W test) and, if the sphericity assumption was violated, Geisser-Greenhouse correction was applied. For the all analysis, the level of significance was set to 0.05, unless stated otherwise.

## Results

### Neuropil staining and morphological aspects of nitrergic neurons in rat primary somatosensory cortex

**Figure 1:**
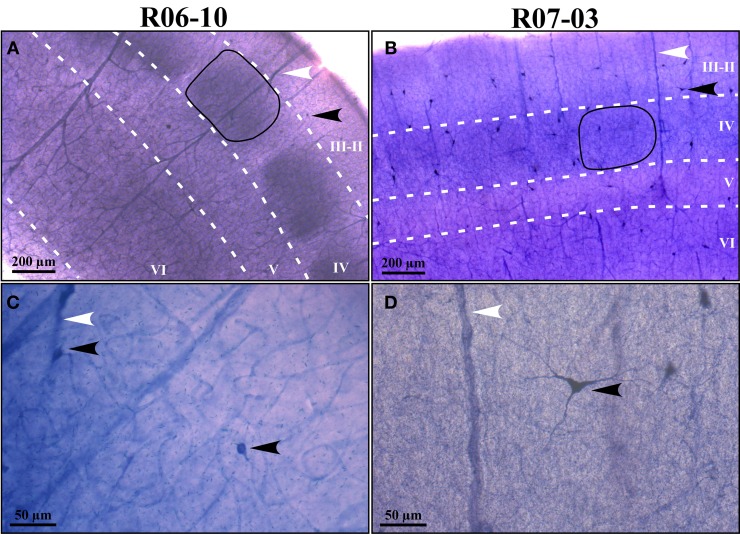
**NADPHd reactive neuropil and nitrergic (NADPHd type 1) neurons. (A,B)** Low-magnification micrographs of cases R06-10 and R07-03, respectively, showing cortical layers (white dashed lines) and barrels in layer IV (the black continuous line depicts one example from each case). Layers IV and VI are more intensely labeled than layers II-III and V. It is not feasible by means of NAPDHd reactivity alone to identify the border between layers II and III. Intensely stained (type 1) NADPHd-reactive neurons can be identified (black arrowheads), and correspond to nitrergic interneurons. Blood vessels are also well-stained (white arrowheads). **(C,D)** Type 1 neurons and blood vessels framed in **(A)** and **(B)** observed in higher magnification (check arrowheads for correspondence). Higher neuropil reactivity in case R06-10 **(A)** provides a better contrast for delimitation of different compartments such as barrels and septa. On the other hand, more intense neuropil reactivity increases background noise making identification of thinner neuritic processes more difficult [such as in **(C)** as compared to **(D)**].

In coronal sections of rat cortex, area S1 was easily identified due to the strong NADPHd-reactive neuropil characteristic of layer IV barrels, intercalated with less reactive septa (Figure 1). This pattern was found in the expected position for the barrel fields (Zilles and Wree, 1985), as previously described with this technique in tangential sections (Franca and Volchan, 1995). Differences in staining intensity of the diffuse NADPHd neuropil label additionally revealed cortical layer borders. Typically, layer I corresponded to a thin densely stained layer close to the cortical surface followed by the less reactive layers II and III. The border between layers II and III could not be reliably identified by means of NADPHd histochemistry. Below the strongly reactive layer IV was the less reactive layer V. Layer VI, although clearly more intensely stained than layer V, was not as dark as layer IV barrels (Figures 1A,B).

**Figure 2:**
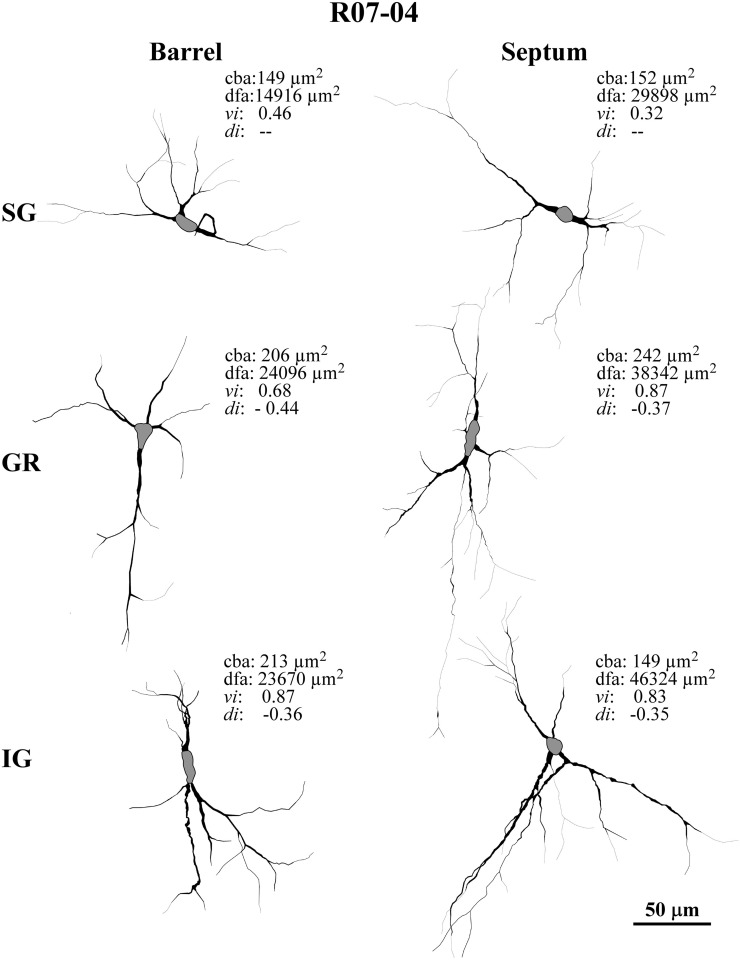
**Examples of typical nitrergic neurons from different cortical compartments in case R07-04 that were 3D-reconstructed in Neurolucida.** All neurons are oriented as if the pia mater were parallel to the top of the figure. Barrel and septal neurons are represented respectively in the left and right columns of the figure. Accordingly, neurons in top, middle, and inferior rows of the figure were respectively located in supragranular (SG), granular (GR), and infragranular (IG) compartments. Neurons in GR and IG layers display more vertically-oriented dendritic trees, while in SG neurons tend to be horizontally-oriented or multipolar. Neurons found in septal columns have a tendency to display more complex dendritic morphology than those located in barrels. Values of four relevant morphological parameters are depicted for each neuron illustrated: cba, cell body area; dfa, dendritic field area; vi, verticality index; di, directionality index. In order to distinguish the limit with primary dendrites, each cell body is represented in gray.

**Figure 3:**
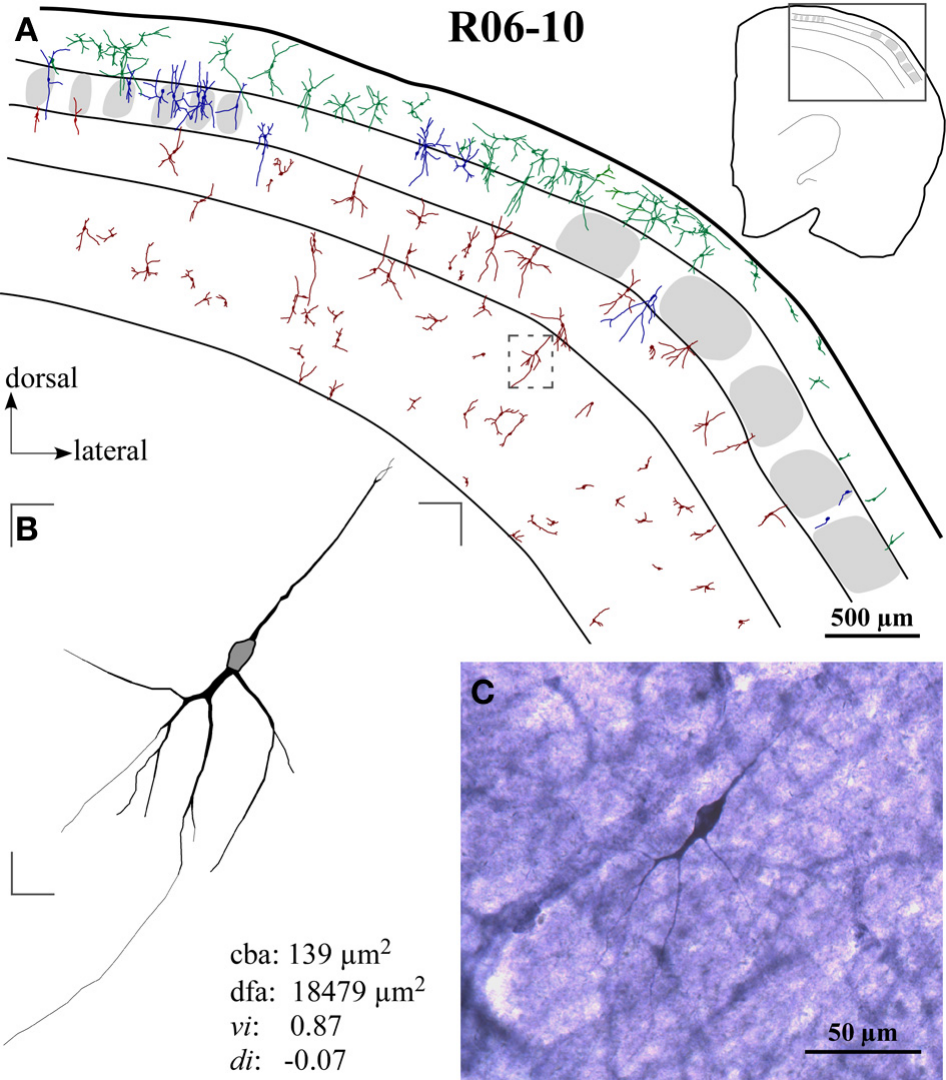
**Case R06-10. (A)** All S1 nitrergic neurons in this 200 μm-coronal section were reconstructed. Neurons with cell bodies located in different cortical layers are represented with different colors. Their spatial distribution does not follow any apparent pattern or regularity. Parts of the section lack the presence of nitrergic neurons while in other parts these neurons are clustered. The absence of barrels in the middle part of this section suggests a cut through two different barrel fields (probably the hindpaw and the postero-medial barrel subfields) separated by the barrel-free dysgranular cortex. Bushy supragranular (SG) neurons observed in this section are not consistently found in other sections of this hemisphere. Nevertheless, they are observed in the other hemispheres analyzed (see Figure 4 for another example). Note that in many instances, dendrites cross over laminar and/or columnar borders. Additionally, some layer IV barrels are devoid of nitrergic neurons, while in others nitrergic cell bodies are localized close to the external border of the barrel. The insert at the upper right corner illustrates the whole section in which a frame depicts the region enlarged in the main part of the figure. A small dashed box frames the neuron illustrated in **(B)**. **(B)** Neurolucida reconstruction of a typical vertically-oriented nitrergic neuron in layer VI. Morphometric parameters depicted for this neuron: cba, cell body area; dfa, dendritic field area; vi, vertically indices; di, directionally indices. Contour of cell body is delimited in gray. **(C)** High-magnification photomicrograph of the same neuron illustrated in **(B)**.

In addition to the conspicuous neuropil staining, NADPHd histochemistry labeled individual neurons with a Golgi-like pattern (Figures 1C,D, 2, and 3B,C). These neurons presented non-pyramidal cell bodies and well-labeled dendritic trees. Because of these features they were identified as type 1 NADPHd neurons (Yan and Garey, 1997). Since all type 1 NADPHd neurons express constitutive NO synthase (Bredt et al., 1991; Dawson et al., 1991; Hope et al., 1991; Matsumoto et al., 1993), these cells will henceforth be referred here as nitrergic neurons, or simply as “neurons.”

**Figure 4:**
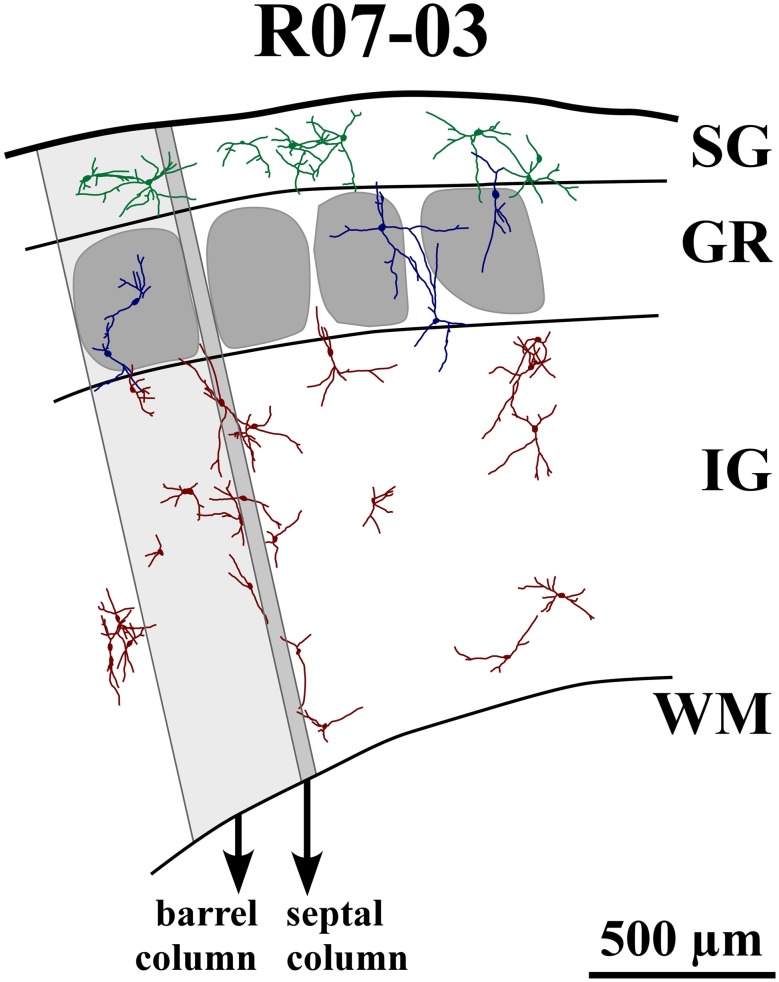
**Reconstruction of part of S1 from case R07-03.** All nitrergic neurons in this part of the tissue are reconstructed and represented. Barrel and septal columns are delimited by lines that are roughly tangential to barrel borders and orthogonal to cortical surface and white matter (WM) boundaries. One barrel column (shaded light gray) and one septal column (shaded dark gray) are depicted for illustration. Neurons from different cortical layers are represented with different colors. Neurons seem randomly distributed with dendrites crossing the limits of barrels and cortical layers. Most neurons in layer IV (GR) and infragranular layers (IG) tend to display vertically-oriented dendritic trees, while in supragranular layers (SG) neurons are either multipolar or horizontally-oriented. Neurons inside layer IV barrels usually have cell bodies located far from the center of the barrel. Their dendritic trees may cross barrel and laminar borders. The same can be observed in the single GR septal neuron represented in this figure. Neurons seem to cluster in pairs or triplets; but, at the same time, leave blank spaces uncovered by their cell bodies and dendritic trees.

Nitrergic neurons presented round or oval cell bodies that gave rise to primary dendrites through an abrupt thinning of the soma profile detected in the same or along different focal planes (Figure 2). Their long dendrites usually ramified two or three times along their path (Figure 2), commonly crossing the limits of different S1 compartments (Figures 3A and 4). Although we used a 100× oil-immersion objective to reconstruct individual neurons, the extent to which a given dendrite could be followed seemed to be inversely proportional to the background activity (Figure 1). Horizontal, vertical, and multipolar nitrergic neurons were present along S1, but vertically-oriented neurons predominated, except in SG layers (Figures 3A and 4, see “Quantitative analysis of morphological parameters of S1 nitrergic neurons.”). In SG layers some nitrergic neurons displayed heavily ramified dendrites, giving them a bushy appearance (Figure 3A). Such SG “arachnoid” neurons were detected in some sections but not in others. They were found in two of the studied hemispheres (Figures 3A and 4).

### Spatial distribution of nitrergic neurons across functional domains of primary somatosensory cortex

**Figure 5:**
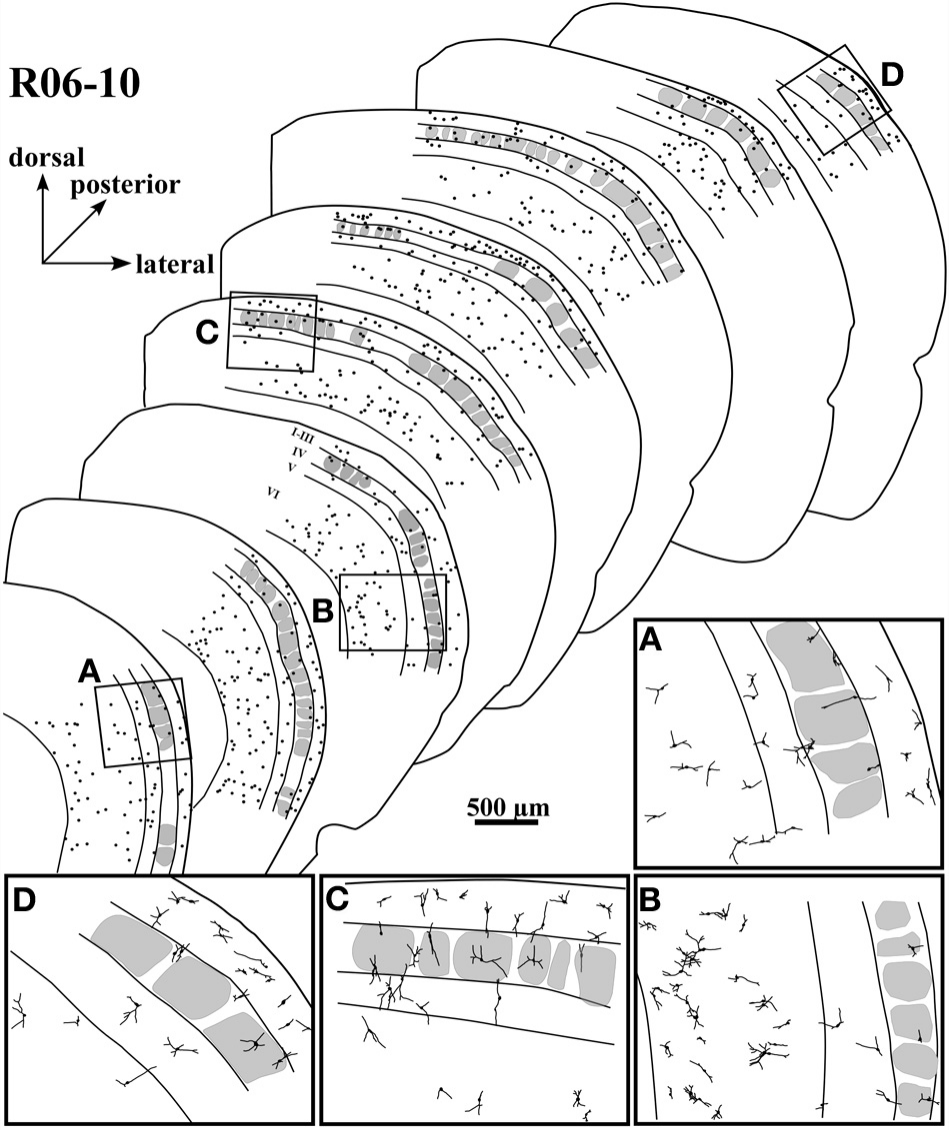
**Spatial distribution of nitrergic neuronal cell bodies in selected sections of hemisphere R06-10, disposed from anterior (lower left) to posterior (upper right).** The four inserts below (from **A** to **D**) correspond to drawings in higher magnification from the respective regions framed in four of the sections. Most of the cells seem to concentrate in supragranular layers and in layer VI.

Nitrergic neurons were detected in all cortical compartments of area S1. They were distributed in an apparently random fashion. In some instances, several neighboring nitrergic neurons were clustered, displaying dendrites seeming in close apposition to each other. However, regions devoid of these cells could also be observed in the same section (Figures 3A and 4). It was apparent by qualitative observation that layers IV and V presented lower nitrergic cell densities than layers VI and SG (Figure 5). Nevertheless, regions in S1 presenting clusters of nitrergic neurons and regions lacking such cells did not alternate with any apparent regularity, nor could be correlated with different cortical compartments in any clear way (Figures 3A, 4, and 5). For instance, although nitrergic neurons were absent from many barrels in layer IV (Figures 3A and 5), they could be found in some other barrels along S1, usually located close to barrel borders (Figures 3A, 4 and 5). Additionally, nitrergic neurons located inside barrels tended to display vertically-oriented dendritic arbors that were usually not constrained by the barrel borders (Figures 3A and 4).

**Figure 6:**
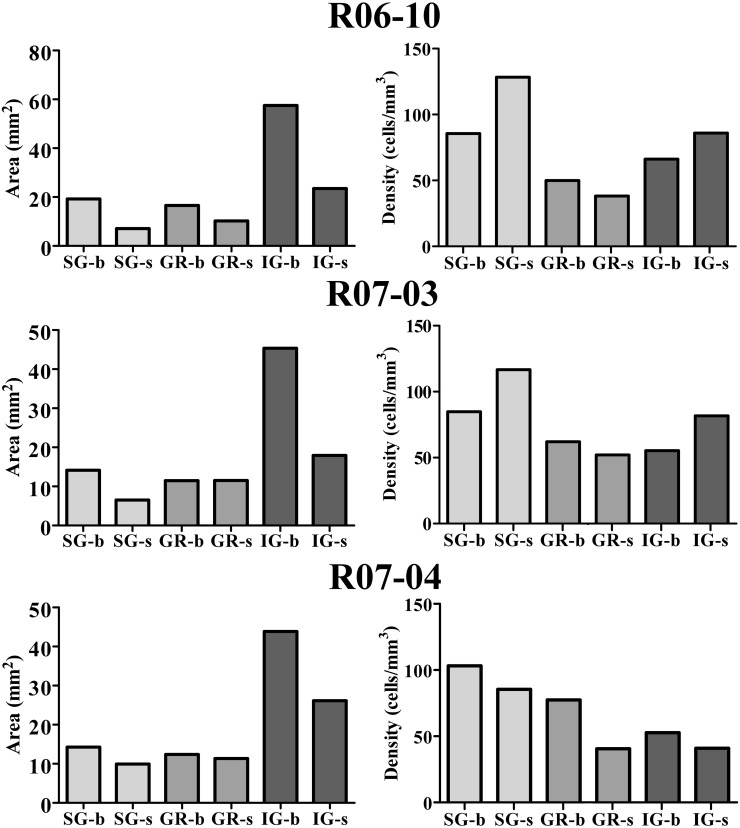
**Cortical compartment area (*left column*) and corresponding nitrergic neuronal density (*right column*) in the three hemispheres studied.** Area measurements were estimated after summing up values obtained in all histological sections obtained in a given hemisphere. Likewise, density was calculated after counting the total number of cell bodies in the same sections. The infragranular layers (IG) correspond to 60% of S1. Barrel columns also correspond to 60% or more of S1. Although IG lodge more than 47% of nitrergic neurons, supragranular layers (SG) present the highest density of nitrergic neurons (about 97 cells/mm^3^ against *circa* 62 cells/mm^3^ in IG, see Table 1). SG-b, supragranular layer at barrel columns; SG-s, supragranular layer at septal columns; GR-b, granular layer at barrel columns; GR-s, granular layer at septal columns; IG-b, infragranular layer at barrel columns; IG-s, infragranular layers at septal column.

In order to quantify the spatial distribution of nitrergic neurons along different cortical compartments of area S1, neuronal density was calculated for S1 in each hemisphere after summing up the number of cell bodies and the area size of individual compartments in all sections throughout S1. Since simple cell counting may result in biased estimations of the number of cellular profiles (Gundersen et al., 1988), adequate stereological corrections were adopted (see “Data extraction and statistical analysis”). Table 1 and Figure 6 present the results of this analysis. In each hemisphere studied, when compartment areas were summed up in all 200 μm-thick sections containing S1 (as if they were positioned side by side), total area of S1 ranged from 107 to 134 mm^2^ (in R07-03 and R06-10, respectively). IG layers constituted 60% of S1 and, correspondingly, contained from 50 to 60% of all S1 nitrergic neurons. Nevertheless, the highest nitrergic neuronal density values were found in SG layers, corresponding to about 97 neurons/mm^3^ (Table 1, Figure 6). These values were 35–100% higher than density measured in IG. The granular layer (GR or layer IV) had the lowest density values, except in case R07-04.

Comparisons between barrel and septal columns (Table 1) revealed that 60–70% of S1 consisted of barrel columns in which approximately 65% of nitrergic neuronal cell bodies were located. However, density in septal columns was approximately 25% higher than in barrel columns, except in case R07-04 in which density measured in barrel columns was similar to that found in the other two hemispheres but 34% higher than that measured in septal columns (Table 1).

### Quantitative analysis of morphological parameters of S1 nitrergic neurons

In hemisphere R06-10 we were able to collect all histological sections along S1 without significant tears or tissue loss. This hemisphere was thus used to perform a complete reconstruction of rat S1, including 3D reconstructions of all nitrergic neurons located in area S1 (*N* = 2125 neurons) as illustrated in Figure 3A and 5. Nevertheless, because many dendrites were cut during tissue sectioning, quantitative parameters were extracted from 180 neurons (30 neurons per cortical compartment) located at mid-section depth and selected based on apparent completeness of all their dendrites. Hemispheres R07-03 (*N* = 180 neurons) and R07-04 (*N* = 180 neurons) also presented a clear neuropil staining pattern, revealing barrels and cortical layers plus well-stained nitrergic neurons that were selected for 3D-reconstruction using the same criteria mentioned above.

Morphological parameters could be either related to the cell body or to the dendritic tree (Table 2). Cell body parameters (cell body area and form factor) displayed mean values that were consistent between hemispheres, with low variation coefficients (up to 26%). However, most of the parameters related to the dendritic tree (*e.g*., dendritic field area, number of segments, etc) displayed large variations in mean values across different hemispheres.

**Table 2:** **Morphometric parameters of nitrergic neurons per hemisphere**.

**Cases**	**R06-10**		**R07-03**		**R07-04**	
**Morphometric parameters**	**Mean**	CV^*^	**Mean**	CV^*^	**Mean**	CV^*^
Cell body area (μm^2^)	145	0.24	180	0.26	165	0.22
Form factor	0.78	0.14	0.81	0.13	0.77	0.15
Number of 1st order dendrites (μm)	3.3	0.29	3.02	0.33	2.96	0.31
Total dendritic length (μm)	504	0.42	1052	0.48	581	0.58
Number of nodes	4.5	0.62	8.77	0.52	7.15	0.58
Number of segments	12.2	0.47	20.52	0.45	17.07	0.50
Fractal dimension	1.00	0.02	1.03	0.02	1.02	0.02
Dendritic field area (μm^2^)	15 × 10^3^	0.69	41 × 10^3^	0.43	20 × 10^3^	2.98
Verticality index	0.71	0.34	0.58	0.54	0.62	0.47

* CV, coefficient of variation.

Morphometry of the reconstructed neurons (Table 2) revealed that the “generic” nitrergic neuron in rat S1 was a non-pyramidal cell (*i.e*., a neuron displaying a rounded cell body with a form factor of 0.8); with a cell body size of 160 μm^2^ from which 2 or 3 primary dendrites emerged. These dendrites tended to ramify once or twice, giving rise to secondary and tertiary dendrites. As depicted above, total DL and dendritic field area measured in the different hemispheres were extremely variable resulting in means ranging, respectively, from 504 to 1053 μm, and from 15 × 10^3^ to 41 × 10^3^ μm^2^.

**Figure 7:**
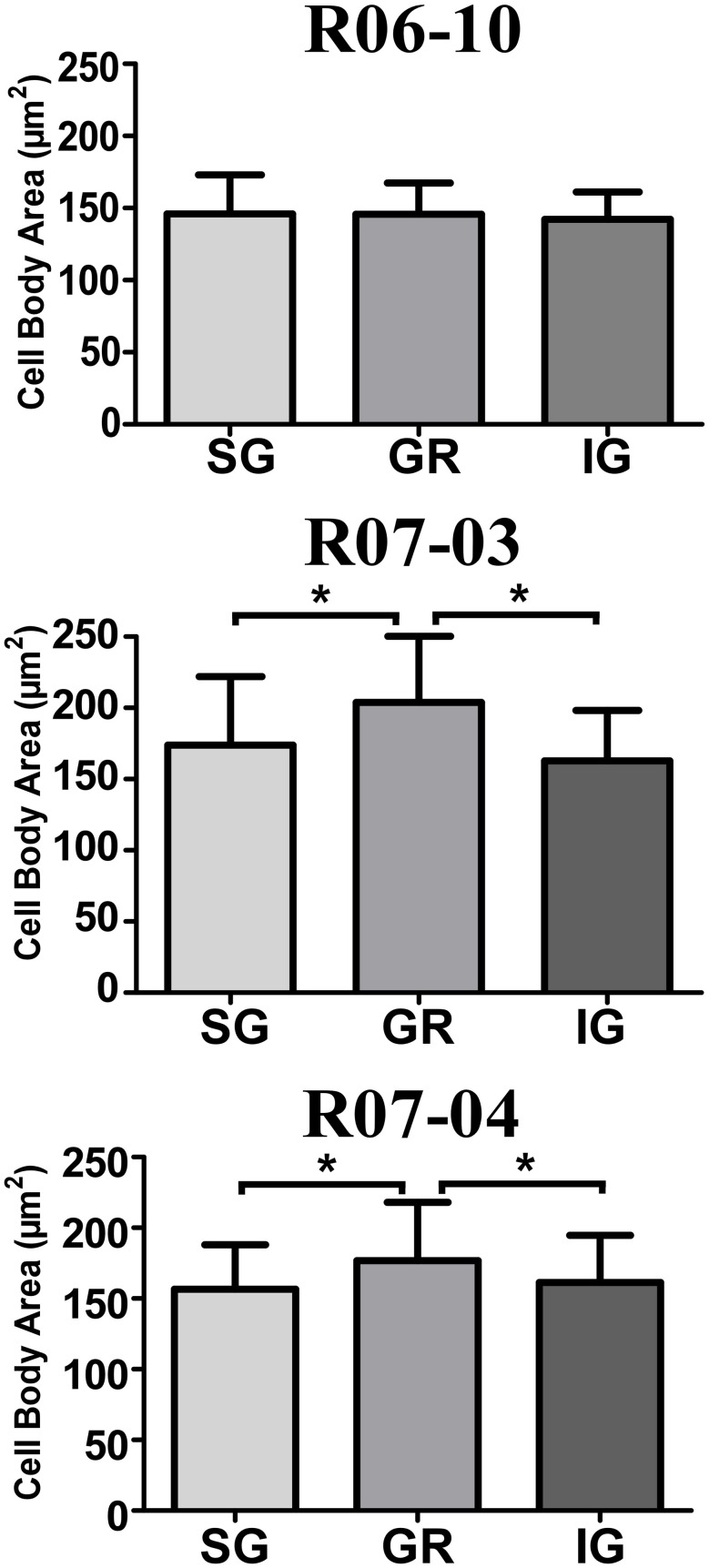
**Mean cell body area of nitrergic neurons.** Layer IV (GR) neurons presented the largest cell body area in cases R07-03 and R07-04. Asterisk (^*^) represents *p* < 0.01.

In hemispheres R07-03 and R07-04, quantitative analysis of cell body area revealed that nitrergic neurons located at layer IV (GR) were 10% larger than those located either in SG or in IG (Figure 7). This difference, although statistically significant (*p* < 0.01), was not noticeable under qualitative inspection. In hemisphere R06-10, no difference in cell body size was detected when neurons from different laminar compartments were compared (Figure 7).

There was no morphological difference when nitrergic neurons from barrel columns were compared with those of septal columns, except for hemisphere R07-04. In this case, DL, number of nodes and segments, dendritic field area, and fractal dimension (a measure of dendritic field complexity) were significantly higher for neurons located in septal columns as compared with those in barrel columns (*p* < 0.01).

### Spatial organization of nitrergic dendritic trees

**Figure 8:**
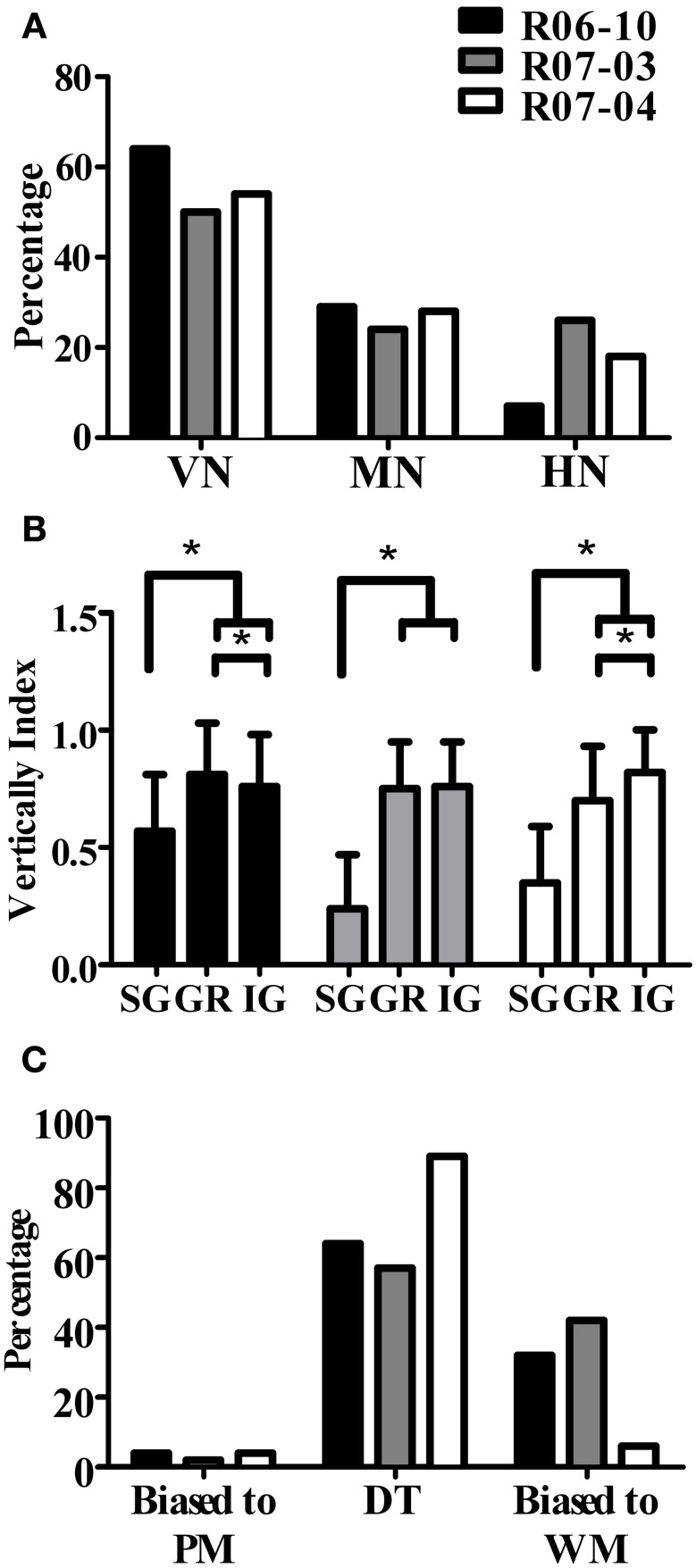
**Quantification of dendritic orientation. (A)** Morphological types of S1 nitrergic neurons classified according to dendritic orientation. Half of these neurons display a vertically-oriented dendritic arbor (VN). The other half of the population is equally represented by multipolar (MN) and horizontally-oriented (HN) neurons. **(B)** Verticality index (*vi*) measured with wedge analysis of 3D-reconstructed S1 nitrergic neurons. Values obtained in supragranular layers (SG) are significantly lower than both granular (GR) and infragranular (IG) layers. This quantitative finding confirms the observation that vertically-oriented nitrergic neurons predominate in GR and IG, while in SG they tend to be horizontal or multipolar. Asterisk (^*^) represents *p* < 0.01. **(C)** Most vertically-oriented neurons in S1 (*i.e*., presenting a verticality index equal or higher than 0.66) display their dendritic trees facing both the pia mater and the white matter, thus corresponding to double-tufted cells (DT). The second most common type of vertically-oriented neurons present a dendritic arbor facing the white matter only. PM: pia mater; WM: white matter.

An important and consistent finding of our study was related to orientation of the dendritic tree, as measured in coronal sections by wedge analysis (see “Morphological parameters analyzed”). In all three hemispheres, 50% or more of the nitrergic neurons presented dendritic trees that were vertically oriented (Figure 8A, Table 3). The other half of this neuronal population was composed of multipolar (ranging from 24 to 29% in R07-03 and R07-04, respectively) or horizontal neurons (from 7 to 26% of the nitrergic neurons in R06-10 and R07-03, respectively, Figure 8A).

**Table 3:** **Distribution of different types of vertically-oriented nitrergic neurons across cortical compartments**.

	**SG-b**^**a**^	**SG-s**	**GR-b**	**GR-s**	**IG-b**	**IG-s**	**Total**^**b**^	**% of total population**^**c**^
**R06-10**								
Vertical cells	11 (9.6%)	12 (10.4%)	23 (20.0%)	25 (21.7%)	25 (21.7%)	19 (16.5%)	115 (100.0%)	63.8
Biased Pia	0 (0.0%)	0 (0.0%)	0 (0.0%)	2 (1.7%)	2 (1.7%)	1 (0.9%)	5 (4.3%)	2.8
DT	5 (4.3%)	9 (7.8%)	15 (13.0%)	14 (12.2%)	14 (12.2%)	16 (13.9%)	73 (63.5%)	40.6
Biased WM	6 (5.2%)	3 (2.6%)	8 (7.0%)	9 (7.8%)	9 (7.8%)	2 (1.7%)	37 (32.2%)	20.6
**R07-03**								
Vertical cells	2 (2.2%)	3 (3.3%)	19 (21.1%)	23 (25.6%)	23 (25.6%)	20 (22.2%)	90 (100.0%)	50.0
Biased Pia	0 (0%)	0 (0%)	0 (0.0%)	0 (0%)	2 (2.2%)	0 (0%)	2 (2.2%)	1.1
DT	0 (0%)	0 (0%)	11 (12.2%)	9 (10.0%)	16 (17.8%)	14 (15.6%)	5 (55.6%)	27.8
Biased WM	2 (2.2%)	3 (3.3%)	8 (8.9%)	14 (15.6%)	5 (5.6%)	6 (6.7%)	38 (42.2%)	21.1
**R07-04**								
Vertical cells	4 (4.3%)	3 (3.2%)	21 (22.3%)	26 (27.7%)	21 (22.3%)	19 (20.2%)	94 (100.0%)	52.2
Biased Pia	0 (0%)	0 (0%)	0 (0.0%)	1 (1.1%)	2 (2.1%)	1 (1.1%)	4 (4.3%)	2.2
DT	4 (4.3%)	3 (3.2%)	20 (21.3%)	21 (22.3%)	18 (19.1%)	18 (19.1%)	84 (89.4%)	46.7
Biased WM	0 (0%)	0 (0%)	1 (1.1%)	4 (4.3%)	1 (1.1%)	0 (0%)	6 (6.4%)	3.3

a Cortical compartments: SG-b, supragranular layer at barrel columns; SG-s, supragranular layer at septal columns; GR-b, granular layer at barrel columns; GR-s granular layer at septal columns; IG-b, infragranular layer at barrel columns; IG-s, infragranular layers at septal columns.

b Number of “vertically-oriented” neurons (i.e., presenting a verticality index higher than 0.65) in a given category. Percentage in parenthesis relates to the number of all vertically-oriented neurons found in the sample.

c Percentage considering the total number of reconstructed neurons in the sample (N = 180 cells per hemisphere).

Multipolar and horizontally-oriented nitrergic neurons were most common in SG. Verticality indices (*vi*) were significantly lower for this laminar compartment than for GR and IG (Figure 8B, *p* < 0.01 for all three hemispheres). Mean *vi* in SG ranged from 0.24 (R07-03) to 0.58 (R06-10), thus lower than the 0.66 threshold used to classify a dendritic tree as vertically-oriented. In GR and IG, mean *vi* ranged from 0.70 (GR in R07-04) to 0.81 (GR in R06-10). This confirmed our qualitative observation of predominance of horizontal and multipolar neurons in SG, whilst vertically-oriented neurons tended to predominate in GR and IG. Vertically-oriented nitrergic neurons were equally distributed in GR and IG, both in barrel and septal columns (Table 3). No significant difference in the orientation of the dendritic tree was detected when neurons from barrel and septal columns were compared against each other in all cases (*p* > 0.05).

Since a vertically-oriented neuron could present its dendritic arbors pointing to different directions, we further calculated the directionality index for nitrergic neurons with *vi* equal or higher than 0.66. In all three hemispheres, 55% or more of the vertically-oriented neurons were double tufted (Figure 8C and Table 3), *i.e*., with dendritic branches extending both toward the white matter and the cortical surface. Moreover, 32% and 42% of the vertically-oriented neurons in hemispheres R06-10 and R07-03, respectively, displayed dendritic trees oriented toward the white matter only (Figure 8C, Table 3).

## Discussion

We described and quantified morphological parameters and the spatial distribution of strongly-reactive (type 1) NADPHd neurons in area S1 of the adult rat. These neurons are known to release NO, an unusual gaseous messenger in the central nervous system (Bredt and Snyder, 1992; Dawson and Snyder, 1994). Based on the pattern of NADPHd neuropil reactivity, we subdivided rat S1 into different vertical and horizontal anatomical compartments typically related to the organizational flow of functional processing in somatosensory cortex.

From qualitative inspection, except for the fact that cortical layers IV and V seemed to present fewer cells, spatial distribution of nitrergic neurons across compartments looked random. Their dendrites were also not necessarily confined inside the cortical compartment in which the parent cell body was located. Nevertheless, density of nitrergic neurons in SG was systematically higher than in other laminar compartments.

We compared morphological parameters from nitrergic neurons reconstructed from different hemispheres and from different compartments in a same hemisphere. Large coefficients of variation were obtained for most parameters, indicating that the morphology of these cells is variable across different animals (Table 2). Additionally, in the three hemispheres analyzed, nitrergic neurons in GR and IG tended to be vertically-oriented, while in SG multipolar, horizontally-, and vertically-oriented neurons were found in equal proportions. Cell body size was larger in layer IV (GR) than in SG and IG in two of these hemispheres. The significance of these results and some considerations about technical factors that might have influenced our results will be discussed below.

### Technical considerations and the variability of morphological parameters

Mean values for morphological parameters of nitrergic neurons were different across analyzed hemispheres (Table 2). For instance, in hemisphere R07-03 mean values for total DL and dendritic field area were about two times larger than those obtained for hemispheres R06-10 and R07-04 (Table 2). A similar trend occurred when cell body area was compared in the three hemispheres. This variability was not due to differences in data collection procedures, since all nitrergic neurons were reconstructed by the same investigator, following the same procedures. Completeness of each reconstructed dendritic tree was further confirmed by a second investigator.

Because aldehyde fixation causes tissue shrinkage, different fixation intensities in the three hemispheres could explain this result. Although we were careful in adopting the same perfusion and fixation procedures in all three animals, variations in fixation intensity are generally very difficult to avoid and might have occurred in our material. This interpretation is supported by the fact that morphological parameters not influenced by tissue shrinkage, such as number of first order dendrites, form factor, verticality index, and fractal dimension, did not vary much.

Although in principle resistant to fixatives such as paraformaldehyde, NAPDH-diaphorase activity of neuronal NOS can be compromised by the intensity of the fixation (Matsumoto et al., 1993; Franca et al., 2000). Thus, it is possible that a stronger fixation may reduce the staining of the dendritic arbor by inactivating histochemical activity of small pockets of NOS contained in thinner processes such as terminal dendrites, which might be more sensible or more exposed to the chemical action of the fixative. In an attempt to circumvent that, longer incubation times are usually performed in order to minimize the risk of not staining the entire dendritic tree. However, longer incubation times result in a darker background that might render thinner processes hard to follow under conventional optical microscopy, thus compromising their reconstruction. We believe that we minimized the possibility of not representing the entire dendritic tree by carefully and systematically reconstructing all neuritic processes using an immersion 100x-objective. Nevertheless, since in hemisphere R07-03 we obtained the largest neurons and the weakest background activity (Figure 1, Table 2), we believe that in some cases very thin terminal dendrites might have been missed due to a darker (noisier) background, or simply because they fell below optical resolution limit (*ca*. 0.25 μm).

Ideally, fixation should be intense enough to render the tissue resistant to damage in histological processing, but light enough to allow histochemical detection of NOS in the most delicate neuronal processes. Assuming a perfect trade-off in fixation intensity, duration of the histochemical reaction is critical. During incubation in NAPDHd histochemical solution, neuronal cell bodies are the first profiles to stain. Primary, secondary, and tertiary dendrites follow suit in this order. Neuropil reactivity with enough contrast to allow identification of the different cortical compartments may be too dark for detection and reconstruction of thinner neuritic processes. Thus, setting the adequate intensity of the NADPHd reaction, either by manipulating intensity of fixation and/or timing of histochemical reaction is difficult to achieve. Probably the best way to optimize NADPHd histochemistry for labeling individual nitrergic neurons is to associate light tissue fixation with short periods of histochemical incubation, thus compromising neuropil reactivity and the identification of cortical compartments.

### Other possible sources of morphological variability of nitrergic neurons

It has been demonstrated that central nervous system expression of “constitutive” NOS corresponds to a plastic phenomenon. In the visual cortex, for instance, NOS expression in neuropil presents a 24 h (circadian) rhythmicity (Hilbig and Punkt, 1997) and can be regulated by visual experience (Aoki et al., 1993).

Experience-dependent regulation of the intracellular distribution of NOS, resulting in variability in the apparent DL and dendritic field size of nitrergic neurons, can occur in the superficial layers of rat superior colliculus (SC) (Tenorio et al., 1998). In the visually-deprived SC, nitrergic activity in distal dendrites diminishes as compared to the contralateral, visually-stimulated, SC. Thus, in the deprived SC, dendritic arbors of nitrergic neurons, as revealed by NAPDHd histochemistry, appear to shrink. This phenomenon corresponds to a subcellular redistribution of NOS, since intracellular injection of fluorescent tracers reveals that these cells have actually the same dendritic field size as those in the visually-stimulated contralateral SC (Tenorio et al., 1998).

Thus, intracellular distribution of NOS can depend on sensory experience, which might vary from animal to animal, possibly resulting in phenotypic variability of nitrergic neuronal morphology such as the one observed in Table 2. In line with this evidence, Romanelli et al. (2007) described a reversible up-regulation of nitrergic expression in rat S1 and other brain regions right after exposure to spatial novelty. In addition, the number and morphological complexity of nitrergic neurons in varying brain regions can also be regulated by numerous factors such as physical exercise (Torres et al., 2006), exposure to light (Chen et al., 2006), and emotional stress (Beijamini and Guimaraes, 2006), which might explain the phenotypic variation of nitrergic neurons obtained in different animals.

### Nitrergic neurons in barrels and septa

When the postero medial barrel field (PMBSF) is analyzed in tangential sections through layer IV, it is clear that nitrergic neurons predominate inside septal as compared to barrel compartments (Valtschanoff et al., 1993; Franca and Volchan, 1995; Freire et al., 2004). According to our previous work in PMBSF (Franca and Volchan, 1995; Freire et al., 2004), the total area occupied by all barrels is equivalent to that of the interbarrel (septal) space, but the number of nitrergic cell bodies located in septal cortex is significantly higher than inside barrels. In our current analysis, when nitrergic neuronal density was calculated for the entire cortical column (summing up neurons and areal sizes of all laminar compartments), we verified in two of the hemispheres examined that density was higher in septal columns than in barrel columns (Table 1).

Curiously, in our material, when we compared the distribution of nitrergic neurons in layer IV only, cellular density is actually higher in barrel compartments in all three hemispheres (Table 1, Figure 6). We believe that a number of factors explain this apparent contradiction with previous findings (Valtschanoff et al., 1993; Franca and Volchan, 1995; Freire et al., 2004, 2005). First, our current analysis was not restricted to the PMBSF (where only the mystacial vibrissae are represented), but encompassed the entire S1, including barrel fields representing other body parts and the dysgranular cortex that separate different barrel fields (for a review see Rice, 1995). This would indicate that rules for distribution of nitrergic neurons vary in different parts of S1, possibly reflecting different cortical circuitries. In line with this hypothesis, recent data suggest that morphology of SG nitrergic neurons actually vary in different parts of primary visual cortex (V1) (Rocha et al., 2012). A second possibility is that we might have overestimated barrel borders, since, due to their oval shapes, they are not as sharply defined in thick coronal sections as in tangential sections through layer IV. Thus, a septal neuron located close to a barrel border might seem inside the barrel when, once observed through a transversal (non-tangential) plane of section, the neuron could be either in front or behind the barrel, but not actually inside it. Support to this notion comes with the fact that we found many cell bodies close to barrel borders. Additionally, different from our previous measurements in tangential sections (Franca and Volchan, 1995; Freire et al., 2004), barrel column total area was larger than that measured in the septal columns (Table 1), further supporting the idea of barrel borders overestimation.

One earlier morphometric analysis of nitrergic neurons in the mouse PMBSF indicated that septal neurons have larger and more complex dendritic fields than nitrergic neurons inside barrels (Freire et al., 2005). Again, this previous study did not encompass the whole S1, and was restricted to neurons located in layer IV. In the present analysis, we used a different species (the rat), and grouped neurons from all cortical layers located either in barrel or septal columns. Using this approach, we did not find morphological differences between neurons located in barrel columns and those of septal columns, except in hemisphere R07-04. In this case, a number of parameters related to dendritic field size and complexity was larger in septal than in barrel nitrergic neurons (*p* < 0.01).

### Nitrergic neuronal morphology and the cortical circuit

We demonstrated that in GR and IG, dendritic trees of nitrergic neurons in rat S1 were predominantly vertically-oriented. Most of these vertically-oriented neurons were either double-tufted or bipolar. In SG, dendritic arbors of nitrergic neurons were equally distributed in horizontal, multipolar, and vertical orientations. Thus, mean verticality index measured in SG was significantly lower than that obtained in GR and IG (Figure 8B, Table 3). Assuming that nitrergic neurons are involved in neurovascular coupling (Drake and Iadecola, 2007; Cauli and Hamel, 2010), non-vertical nitrergic processes in SG could act in concert to maintain the metabolic demands necessary for horizontal integration, while in GR and IG nitrergic neurons would simultaneously keep activity vertically restricted to the cortical column. However, important lateral transcolumnar projections occur in GR and IG of rat S1 (Schubert et al., 2007). In addition, nitrergic neurons are not necessarily confined to the anatomically-defined cortical compartments. Similar dendritic patterns have been described for pyramidal neurons located close to the borders of cytochrome oxidase blobs in V1 (Hubener and Bolz, 1992; Malach, 1992). These findings give support to the idea that cortical processing is not as strictly compartmentalized in vertical columns (Swindale, 1990; Horton and Adams, 2005; Rockland, 2010) as previously suggested (Mountcastle, 1997; Douglas and Martin, 2004).

We additionally observed that in two of our hemispheres (R07-03 and R07-04) cell body size was larger in nitrergic neurons located inside layer IV than in the other laminar compartments (*i.e*., SG and IG). Functional significance of this finding is uncertain. Nitrergic cell bodies can be viewed as nodes for NO release. In principle, these larger cell bodies may release larger amounts of NO per neuron, in the same cortical layer (*i.e*., GR) that presented the lowest numbers of nitrergic neurons (Table 1). On the flip side, it is already well-documented that, in primary sensory areas such as S1, GR has a high metabolic activity, as identified by intense histochemical reactivity for enzymes such as succinate deshydrogenase and cytochrome oxidase (Wong-Riley and Welt, 1980; Wallace, 1987; Riddle et al., 1993). This high concentration of metabolic enzymes in layer IV correlates with an intense reactivity to NAPDHd and NOS (Wong-Riley and Welt, 1980; Aoki et al., 1993; Wong-Riley et al., 1998), suggesting that NO synthase expression is coupled to neural activity, perhaps through a positive feedback loop in which NO acts as a retrograde messenger (Garthwaite and Boulton, 1995). NO can thus be released either through “large” cellular profiles such as neuronal cell bodies and dendrites, or through the fine processes (represented by axons, axon terminals, and terminal dendrites) that comprise the reactive neuropil. The precise role played by these two sources of NO should be relevant for cortical physiology, and needs to be further investigated.

### Conflict of interest statement

The authors declare that the research was conducted in the absence of any commercial or financial relationships that could be construed as a potential conflict of interest.
